# Initial clinical experience with a novel mechanical thrombectomy device-the ThrombX retriever

**DOI:** 10.1177/15910199221118146

**Published:** 2022-09-15

**Authors:** Daniel Behme, Martin Wiesmann, Omid Nikoubashman, Hani Ridwan, Dimah Hassan, Thomas Liebig, Christoph Trumm, Markus Holtmannspötter, Istvan Szikora

**Affiliations:** 1 Department of Neuroradiology, 39067 Otto-von-Guericke University Clinic, Magdeburg, Germany; 2 Department of Neuroradiology, 39058 University Hospital RWTH, Aachen, Germany; 3 Department of Neuroradiology, 9183 Ludwig Maximilian University Hospital, Munich, Germany; 4 Department of Neuroradiology, 9211 Nuremburg Clinic South, Paracelsus Medizinische Privatuniversität (PMU), Nuremberg, Germany; 5 Department of Neurointerventions, National Institute of Mental Health, 569121 Neurology and Neurosurgery, Budapest, Hungary

**Keywords:** Thrombectomy, new device, stent retriever

## Abstract

**Background and Purpose:**

The ThrombX Retriever is a novel mechanical thrombectomy device that adjusts the distance between two mesh segments to axially hold thrombus. A post-market study assessed safety and performance in acute ischemic stroke patients with large artery occlusion.

**Methods:**

A single-arm prospective multi-center study enrolled patients at 5 European Centers. Patients had a symptomatic large-artery occlusion of the intracranial Internal Carotid or the Middle Cerebral Artery, M1 segment. The primary outcome measure was the modified treatment in cerebral infarction (mTICI) score, on the immediate post-procedure angiogram after up to three device passes. Key secondary outcome measures were the mTICI score after a single pass and functional independence, defined as an mRS score ≤ 2 at 90 days.

**Results:**

Thirty patients (16 Females, mean age 72 years), with NIHSS 4-25 (mean 15.5) were treated. Twenty-eight (93%) achieved mTICI 2b-3 within 3 passes, and 24 (80%) were with the first pass (FP). FP mTICI 2c-3 reperfusion was achieved in 19 (63%) cases. Seventeen of 24 (71%) patients treated with a balloon guide and the ThrombX Retriever had a FP mTICI 2c-3 reperfusion. After all interventions, mTICI 2b-3 was seen in 30 (100%) patients. Twenty-one of the 29 (73%) patients with 90-day follow-up were functionally independent (mRS≤2). No device-related serious adverse events were observed.

**Conclusion:**

This preliminary study suggests the ThrombX Retriever is safe and has a high rate of substantial reperfusion. A larger prospective trial to assess the device effectiveness is planned.

## Introduction

Endovascular thrombectomy has been shown to improve outcome for acute ischemic stroke patients with large artery occlusions of the anterior cerebral circulation.^1^ Achieving a First Pass Effect (FPE), when defined as complete reperfusion (TICI 3) with one pass of a thrombectomy device, has been shown to improve clinical outcome.^2,3^ Subsequent passes are associated with a decreased likelihood of good neurological outcome.^4,5^ Near-complete reperfusion (TICI 2c) also has been demonstrated to provide similar rates of good clinical outcome when compared to TICI 3 reperfusion.^6^ A current metanalysis including thirteen thrombectomy studies of 4197 patients reported a First Pass TICI 2c-3 rate of 32%.^4^ Prospective studies evaluating newer mechanical thrombectomy devices have shown First Pass TICI 2c- 3 rates of 40.1 and 41.8%.^7,8^ These relatively low rates of First Pass TICI 2c-3 signal a need for a device with improved TICI 2c-3 First Pass reperfusion.

One of the determinants of mechanical reperfusion has been shown to be clot composition, with fibrin rich thrombi demonstrating increased number of passes and longer procedure times when compared to erythrocyte rich thrombi.^9–12^ We evaluated a CE Marked mechanical thrombectomy device in a prospective multi-center single-armed study. The device is a novel mechanical thrombectomy device that allows the physician to adjust the distance between two mesh baskets to axially engage and hold thrombus. Preclinical evaluation of the device suggest that it has high rates of first pass recanalization regardless of clot composition.^13^ This study was performed to obtain preliminary safety and performance data and understand the effect of this novel device on first pass reperfusion.

## Methods

Thirty patients were enrolled in a prospective study at 5 European Centers from July 2020 to November 2021, to obtain a post market assessment of the safety and performance of the ThrombX Retriever in the revascularization of patients presenting with acute ischemic stroke (AIS) secondary to intracranial, large vessel occlusion. Patients with AIS due to an angiographically proven occlusion (mTICI, 0–1) in the internal carotid and middle cerebral artery M1 segment were eligible for enrollment. Patients were included if their pre-stroke modified Rankin Scale (mRS) was ≤2, the baseline National Institutes of Health stroke scale (NIHSS) score was ≥4 and ≤25, and the Alberta Stroke Program Early Computed Tomography (ASPECT) score was ≥6 or the core infarct volume was <50 mL on magnetic resonance imaging. Key exclusion criteria included: Presence of carotid dissection, imaging or clinical evidence of concomitant posterior circulation stroke or acute stroke involving both anterior circulations, CT or MRI evidence of hemorrhage, mass effect, or evidence of internal carotid artery tandem stenosis that was flow limiting.

The study protocol was approved by the ethics committee at each participating site. All patients or their legally authorized representative provided written informed consent before enrollment.

Physicians used standard of care techniques for catheterization of the cerebral circulation with fluoroscopic guidance and patient sedation. All the patients in this study were treated using femoral artery access. The ThrombX Retriever was used with standard commercially available distal access or aspiration catheters, balloon guide catheters, and guide catheters, that have CE Mark approval for access to the extracranial and intracranial circulations and are used as adjunctive devices for intracranial thrombectomy. This included 8F conventional guide catheters (6 cases) or balloon guide catheters (24 cases) in the extracranial circulation and 5F or 6F distal access or aspiration catheters in the intracranial circulation (15 cases). In 15 cases the ThrombX Retriever was used with a balloon guide alone.

The ThrombX Retriever was used in accordance with the Instructions for Use (see supplementary material). Up to 3 passes of the ThrombX Retriever were allowed to achieve complete reperfusion (mTICI 3). However, the treating physician could use an alternative mechanical thrombectomy device, or suction thrombectomy catheter at their discretion at any time after an initial pass with the ThrombX Retriever.

**Figure 1.**
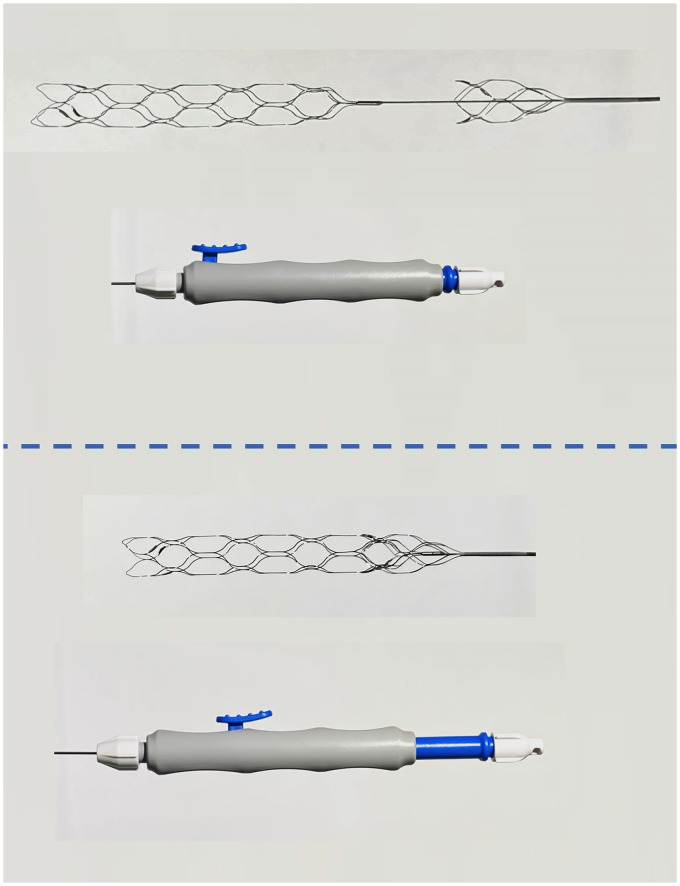
Thrombx retriever with external handle. Top images show handle with proximal and distal baskets separated by 10 mm gap. Lower image shows handle with thumb slide retracted and baskets fully opposed.

**Figure 2.**
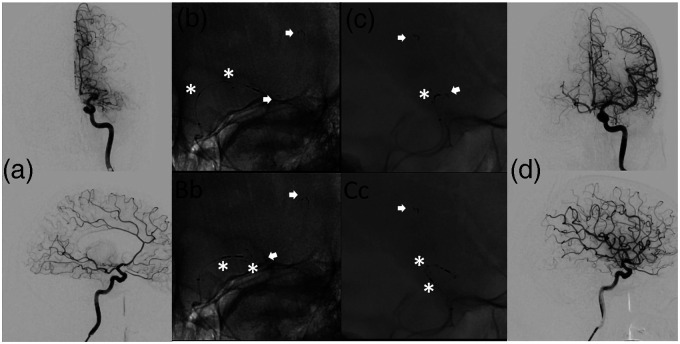
Exemplary case. a ap and lateral DSA images showing a proximal MCA M1 occlusion of the left side. b (Bb) and c (Cc) showing ap and lateral images of the unconstrained TXRL, arrows indicating distal basket markers, asterisks indicating proximal device and basket markers. d showing final complete reperfusion success after one pass.

The ThrombX Retriever is a novel mechanical thrombectomy device that allows the physician to adjust the distance between two mesh nitinol segments or baskets to axially engage and hold thrombus (Figures 1 and 2). Two sizes of the ThrombX Retriever are available, one with the distal basket length having a 20 mm working length in the shorter version (TXR) and a 30 mm working length in the longer version (TXRL). The other dimensions in the two versions were identical. Proximal and distal baskets are 4.5 mm diameter and both versions have a 10 mm gap between baskets with the proximal mesh basket having a working length of 8 mm. When the study was initiated, the device was cleared for use in an .027-inch microcatheter, and subsequently the device was cleared for an .021-inch microcatheter. The device is introduced with the baskets separated. The front of the proximal basket is positioned at the proximal face of the clot so that thrombus is in the intervening space between the baskets. The baskets are then brought together to axially engage the thrombus (Figure 1 and 2). This is accomplished by depressing the thumb slide on the handle to unlock the baskets and sliding the thumb slide back to move the distal basket into the proximal basket.

Prior to thrombectomy, digital subtraction angiography was performed to define the angiographic architecture of the occluded vascular segment. The mTICI score was recorded from each attempt and at the completion of the procedure by the local investigator. Angiographic success was defined as achieving thrombolysis in cerebral infarction (mTICI) grade 2b (50–89% reperfusion), mTICI 2c (90–99% reperfusion), or mTICI 3 (100% reperfusion). Clinical assessments were performed by an independent neurologist, using the National Institutes of Health Stroke Scale (NIHSS) and modified Rankin scale (mRs) were performed at 5–7 days post-procedure or discharge, whichever came first, as well as at 30 days ( + /- 5days) and 90 days ( + /-14 days).

Computed tomography (CT) imaging was obtained 24 h (+/- 6 h) post-procedure to assess for any hemorrhage. Hemorrhage was assessed as symptomatic if there was an intracerebral hemorrhage associated with a neurologic deterioration of ≥ 4 points on the NIHSS score from the baseline score, or it was assessed as asymptomatic.

Primary effectiveness was judged by the ability of the device to achieve ≥ mTICI 2b in the target vessel after three or fewer passes of the device and before use of an adjunctive thrombectomy device. A key secondary effectiveness measure was the mTICI 2c-3 fist pass reperfusion. Key secondary outcome measures included: Functional independence, defined as an mRS score ≤ 2 at 90 days, embolization in a previously uninvolved territory on angiogram. incidence of device-related serious adverse events, symptomatic and asymptomatic intracranial hemorrhage, procedure related mortality rate at 7 days and all-cause mortality at 90 days.

Demographics and baseline characteristics are summarized using descriptive statistics. Standard descriptive statistics for categorical endpoints are the number and percent of patients with each level of the endpoint. For numeric endpoints, the standard descriptive statistics include the number of non-missing observations (n), the mean and standard deviation (SD), or median and interquartile range (IQR).

## Results

Patient baseline characteristics for the 30 patients enrolled in the study are summarized in Table 1. The mean age was 71.8 years, 16 (53%) were female, and the median (interquartile range [IQR]) baseline NIHSS was 15.5 (11–19.75). The median baseline ASPECT score was 8 (IQR, 8–9). The MCA M1 segment was the site of occlusion in 93% of the cases and the remaining 7% of cases were in the internal carotid artery.

**Table 1.** Baseline characteristics of patients.

Characteristic	N = 30
Age, range (mean ± SD)	42–85 years (71.8 ± 11.7)
Female, n (%)	16 (53%)
NIHSS score at baseline, median (IQR)	15.5 (11–19.75)
Medical history, n (%)	
Hypertension	25 (83%)
Smoking History	7 (23%)
Diabetes mellitus	8 (27%)
Atrial fibrillation	11 (37%)
Dyslipidemia	6 (20%)
History MI/CAD/Angina	4 (13%)
Previous Stroke	4 (13%)
Occlusion Site, n (%)	
Internal Carotid Artery	2 (7%)
Middle Cererbral Artery M1 segment	28 (93%)
CT ASPECT Score, Median (IQR)	8 (8,9)
Reperfusion Occlusion side left, n (%)	13 (43%)
Symptom onset to arterial puncture, minutes; median (IQR)	184 (124–242)

Values are mean (SD), median (IQR), or n (%). ASPECT: Alberta Stroke Program Early CT Score for MCA territory stroke; CT: computed tomography; IQR: interquartile range; NIHSS: National Institutes of Health Stroke Scale.

The median time from symptom onset to puncture was 184 min (IQR, 124–242). Four patients (13%) received intravenous tPA before endovascular therapy. A balloon guide catheter (BGC) was used in 80% of cases (50% BGC alone, 30% BGC plus distal aspiration catheter).

**Figure 3.**
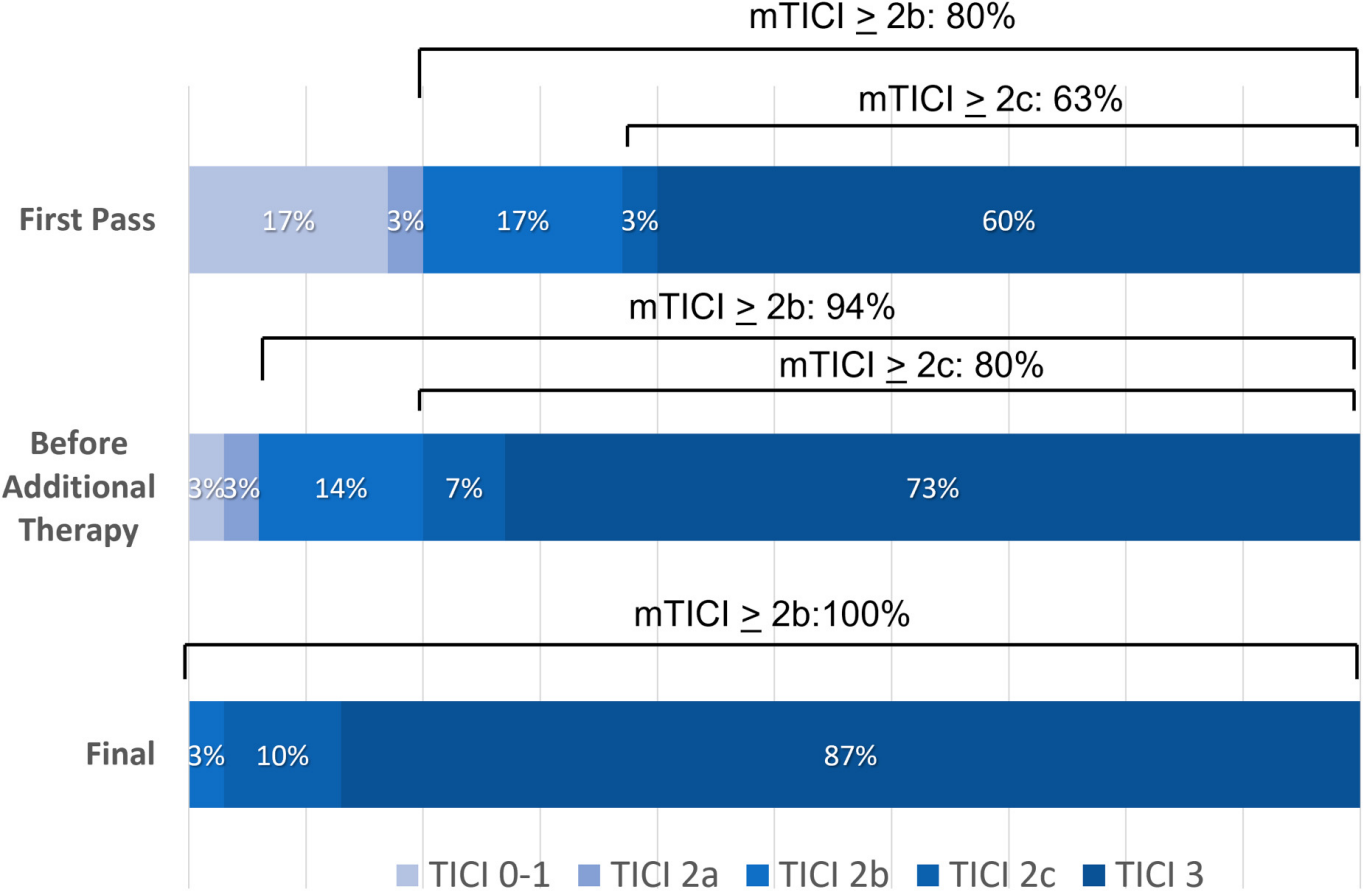
Reperfusion as measured by the mTICI score after first pass of ThrombX retriever, after up to 3 passes, and after additional therapy.

The ThrombX Retriever achieved successful reperfusion (mTICI 2b-3) within 3 passes before additional therapy in 28 of 30 cases (93%). In the two cases that had additional therapy before a mTICI 2b-3 reperfusion was achieved, one patient was TICI 0 and one patient was TICI 2a. The rate of mTICI ≥2c reperfusion within 3 passes of the ThrombX Retriever was 80%. Figure 3 shows the complete distribution of perfusion outcomes. FP effect (defined as mTICI ≥2c after one pass) occurred in 19 (63%) patients and modified FP effect (mTICI ≥2b after a one pass) was seen in 24 (80%) patients. In the patients treated with a balloon guide the FP effect was seen in 17 of 24 patients (71%) overall and 12 of the 15 patients (80%) treated with the ThrombX Retriever and a BGC alone.

Median treatment time from puncture to reperfusion (mTICI ≥ 2b) was 22.5 min (IQR, 16.5–36). The mean number of passes ± Standard deviation (SD) was 1.5 ± 0.9.

Additional therapy was used in 6 cases (20%). In 4 cases a 3 mm stent retriever was used to clear a more distal occlusion after a mTICI 2b reperfusion was achieved with the ThrombX Retriever. In one case a 5F aspiration catheter was used after a mTICI 2a reperfusion had been achieved with a single pass of the ThrombX Retriever. In one case the ThrombX retriever was used for an initial unsuccessful reperfusion, subsequently 3 other passes were performed with other stent retrievers that also proved unsuccessful and a fifth pass was performed with the ThrombX Retriever resulting in a TICI 3 reperfusion.

**Figure 4.**
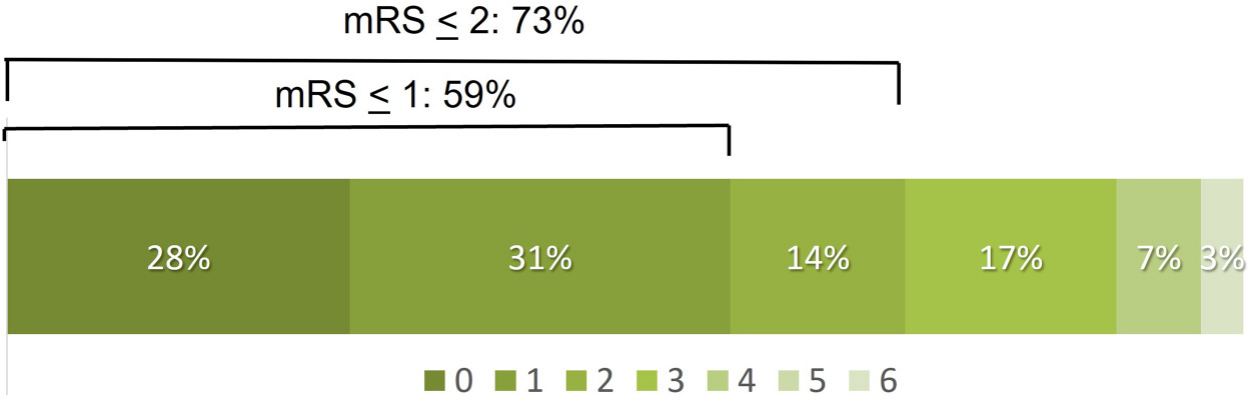
Distribution of 90-day clinical outcomes on the modified Rankin Scale (mRS). mRS was available from 28 of 30 patients.

The median 24-h NIHSS score was 4 (IQR 1.5–7), which represented an average decrease of 11 points compared to the pre-treatment score. Functional independence (mRS 0–2) was attained in 21 of 29 patients (73%) with 90-day follow-up. Figure 4 shows the 90-day outcome distribution n for the modified Rankin Scale scores. There was one death in the cohort from an unknown cause which occurred 45 days after the stroke.

There were no symptomatic intracranial hemorrhages observed. Three patients (10%) had asymptomatic hemorrhages, two in the territory of infarction and one in another territory (in the posterior circulation). Two of these patients had improved NIHSS scores and one showed no change. No embolization into a new territory was observed. Vasospasm, which resolved, was reported in three cases. There were no serious device or procedure related adverse events reported.

## Discussion

The ThrombX Retriever achieved high rates of reperfusion in this prospective, multicenter evaluation, with TICI 2b-3 reperfusion attained in 93% patients and TICI 2c-3 reperfusion in 80%. This was accompanied by a high rate of favorable clinical outcomes, with 73% of patients demonstrating functional independence (mRS 0-2) at 90 days.

First pass effect, which can be defined as achieving mTICI 2c-3 reperfusion with one pass, has been shown to produce better patient outcomes and is a more recent measure to assess thrombectomy device effectiveness.^2^ The ThrombX Retriever achieved a FPE in 63% of patients, which compares favorably to rates of FPE reported in recent thrombectomy device studies such as the ARISE II study, reporting a 40% FPE^8^ and the TIGER trial, demonstrating a 41.7% FPE.^8^

Conventional Stent Retrievers are widely used in combination with distal aspiration catheters (DAC) and BGC. The DAC when used with the conventional stent retriever is placed at the clot face to provide proximal control of the thrombus and improve clot retention.^14–16^ The use of proximal flow control during the thrombectomy procedure with a BGC in the cervical segment of the ischemic circulation has also been shown to improve FPE and clinical outcomes.^17–20^ Seventeen of the 24 patients (71%) in this study with BGC use had a FPE. The unique design of the ThrombX Retriever allows for capture of the thrombus between the distal and proximal components and promotes proximal control of the clot. This may allow users to simplify the thrombectomy procedure by eliminating the need for the DAC. Indeed 12 of the 15 patients (80%) treated with the ThrombX Retriever and a BGC alone had a FPE.

The median time from puncture to achieving TICI 2b-3 reperfusion was 22.5 min (IQR, 16.5–36), which compares favorably to the median times reported for the Embotrap in the ARISE II study (35 min; IQR, 24–58)[7], the Tigertriever in the TIGER trial (25 min; IQR 17–43)[8]. This may partly be a function of the higher first pass rates observed, but it indicates that the extra maneuver to capture the thrombus between the device components prior to withdrawal, does not add significant procedure time.

Clinical outcomes in the present study were favorable. Functional independence (mRS score 0–2) at 90 days was achieved in 73% of patients. This rate of good functional outcome is likely related to the rapid reperfusion seen with the ThrombX Retriever. In this initial study, the safety profile of the device appears to be acceptable as well. There were no symptomatic hemorrhages and no device related or procedure related serious adverse events reported.

The study has limitations. The small number of patients and investigator adjudicated reperfusion data are reasonable criticisms of the study. However, the positive results observed in this prospective, multi-center study using ThrombX Retriever as the primary device, warrant further investigation in a larger study.

## Supplemental Material

sj-pdf-1-ine-10.1177_15910199221118146 - Supplemental material for Initial clinical experience with a novel mechanical thrombectomy device-the ThrombX retriever Supplemental material, sj-pdf-1-ine-10.1177_15910199221118146 for Outcome prediction value of critical area perfusion score for acute basilar artery occlusion by Daniel Behme, Martin Wiesmann, Omid Nikoubashman, Hani Ridwan, Dimah Hassan, Thomas Liebig, Christoph Trumm, Markus Holtmannspötter and Istvan Szikora in Interventional Neuroradiology
