# Supplementary material for: Initial clinical experience with a novel mechanical thrombectomy device-the ThrombX retriever
Source: Interv Neuroradiol. 2022 Sep 15;30(2):183–8. doi: 10.1177/15910199221118146 (PMC11095356; doi:10.1177/15910199221118146)
Supplement: sj-pdf-1-ine-10.1177_15910199221118146 - Supplemental material for Initial clinical experience with a novel mechanical thrombectomy device-the ThrombX retriever [file sj-pdf-1-ine-10.1177_15910199221118146.pdf]

INSTRUCTIONS FOR USE

DEVICE DESCRIPTION

The ThrombX Retriever consists of two self-expanding nitinol baskets independently attached to a hypotube (proximal) and a core wire (distal) (**Figure 1**). The device is available in four configurations, the TXR, TXR021, TXRL, and TXRL021. Platinum markers on the proximal and distal baskets allow fluoroscopic visualization. Under fluoroscopy, the basket markers appear thinner than the thicker marker on the core wire (behind distal basket) and the hypotube (behind proximal basket). An introducer sheath that is slid over the baskets facilitates loading of the device into the proximal hub of a microcatheter.

Figure 1: Distal Basket and Proximal Basket

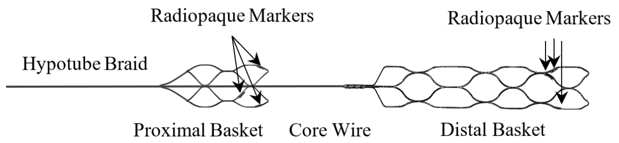

A handle on the proximal end of the device keeps the baskets locked in position while advancing the device in the microcatheter (**Figure 2**). Once the device is deployed, the handle is used to control the distance between baskets. A distal cap on the handle is tightened on the hypotube that is connected to the proximal basket. A proximal cap on the handle is tightened on the inner core wire that is connected to the distal basket. A window in the proximal cap confirms that the inner core wire is present. A thumb slide is pressed to unlock the handle and retract the distal basket. Once unlocked, the thumb slide is pulled proximally to withdraw the distal basket and narrow the gap between baskets.

Figure 2: Deployment Handle

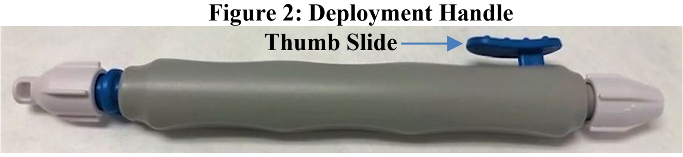

The ThrombX Retriever is compatible with 0.021 in (0.053 mm: TXR021, TXRL021) or 0.027 in (0.69 mm: TXR, TXRL) inner diameter microcatheters. Refer to the appropriate microcatheter and guide catheter manufacturer's *Instructions for Use* for microcatheter and guide catheter compatibilities.

INTENDED USE

The ThrombX Retriever is intended to restore blood flow in patients with acute ischemic stroke secondary to intracranial occlusive vessel disease.

CONTRAINDICATIONS

This device is contraindicated in patients with a known hypersensitivity or allergy to nitinol.

WARNINGS

- Do not use the device in extremely tortuous or delicate vessels that may prevent safe access of the device
- If excessive resistance is felt at any time during use, stop the procedure and determine the cause before proceeding. If the ThrombX Retriever is unable to cross the occlusion, discontinue use.
- If there is excessive resistance while withdrawing the tip of the ThrombX Retriever into the guide catheter, then remove the guide catheter and ThrombX Retriever *together as a unit* to the introducer sheath.
- The use of the ThrombX Retriever is recommended only at hospitals specialized in endovascular treatment of neurovascular diseases where high quality fluoroscopy equipment is available and where appropriate facilities are available for managing potential complications of the procedure.
- Only physicians thoroughly trained and educated in endovascular treatment of intracranial occlusive disease should use this device.

PRECAUTIONS

- Inspect the sterile package carefully. Do not use if:
  - The package or seal appears damaged
  - Contents appear damaged
  - The expiration date has passed
- The ThrombX Retriever is intended for SINGLE USE ONLY; DO NOT RESTERILIZE. Use aseptic technique in all phases of handling. Discard the device after one procedure.
- Store in a cool, dry place.
- DO NOT autoclave.
- DO NOT attempt to load device into microcatheter without introducer sheath.
- Maintain a constant flush infusion between the microcatheter and the ThrombX Retriever or guidewire.
- Do NOT attempt to torque, turn, or rotate the ThrombX Retriever.
- Dispose of all used devices in accordance with hospital policy for biohazardous materials.

ADVERSE EVENTS

Potential complications include, but are not limited to:

- Death
- Transient and permanent neurological deficit (stroke)
- Blindness
- Nerve injury
- Compression
- Irritation
- Cerebral ischemia
- Acute myocardial infarction
- Cardiac dysrhythmia
- Arteriovenous fistula
- Cerebral edema
- Dissection or Perforation
- Continued occlusion
- Hemorrhage, hematoma
- Respiratory complications, pneumonia
- Device failure events (failure to deploy, strut fracture, detachment)
- Pseudoaneurysm formation
- Vasospasm
- Hypotension
- Air embolism
- Distal thrombus embolization
- Recurrence of thrombus
- Allergic reactions to device, drugs, or contrast medium
- Infection
- Sepsis
- Renal insufficiency and/or failure

PROCEDURE

ThrombX Retriever Preparation and Microcatheter Hub Insertion

1. Maintain flush through the guiding catheter and microcatheter per standard endovascular practice. Position the microcatheter beyond the proximal face of the clot using standard endovascular technique as shown in **Figure 3**.
2. Remove the ThrombX Retriever from the pouch and flush through the Adapter (attached to the inner hoop) with heparinized saline while in the hoop. Keep hydrated.
3. Remove the Adapter from the hoop and place within the sterile field.
4. Detach the handle of the ThrombX Retriever from the packaging card clips by moving the handle upward to remove from the packaging clip and withdraw the device straight out from the hoop.
5. Open the Rotating Hemostatic Valve (RHV) on the back end of the microcatheter and advance the introducer through the RHV with<u>out</u> seating the introducer into the microcatheter hub.
6. Tighten the RHV onto the introducer to prevent back bleeding, but not so tightly as to damage the ThrombX Retriever hypotube contained inside.
7. Flush by infusing heparinized saline into the microcatheter via the RHV side port until fluid is observed exiting the proximal end of the introducer, which confirms the ThrombX Retriever is flushed.
8. Open the RHV. Advance the Introducer until the tip of the introducer is tightly lodged in the microcatheter hub. Tighten the RHV. The device is ready to be advanced up the microcatheter.

Delivery and Deployment

1. Advance the ThrombX Retriever through the microcatheter with short strokes while observing the hub to ensure the baskets pass safely. Advance until the handle reaches the proximal end of the introducer.
2. Pull the proximal end of the Introducer laterally (5 to 10 cm) to open the Introducer slit.
3. Open the RHV, slide the Introducer out of the RHV, and peel the Introducer off the device by continuing to pull it back (away from the Handle). Remove Introducer completely and place on sterile surface.

4. Advance the device toward the distal end of the microcatheter using fluoroscopy.

Figure 3: Microcatheter Placement

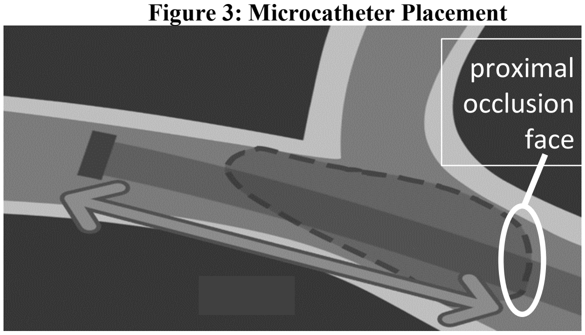

Note: Place the microcatheter per standard techniques with the tip approximately 3 cm (TXR) or 4 cm (TXRL) beyond the proximal face of the occlusion.

5. Confirm the microcatheter tip is 3 cm (TXR) or 4 cm (TXRL) beyond the proximal face of the occlusion. Advance the device while in the microcatheter so that the large proximal "wire" marker of the distal basket is beyond the proximal face of the occlusion and the distal markers of the proximal basket are at the face of the occlusion. The proximal end of the distal basket will be within the clot.

Warning: If resistance is felt **during** advancement, stop and determine the cause of resistance before proceeding.

6. To deploy the baskets, maintain device position by stabilizing the hypotube. At the same time, gently pull back on the microcatheter. As the microcatheter is pulled back, the distal basket will be deployed (basket markers visible on fluoroscopy), and the proximal basket will open at the clot face.

Caution: Do not torque, turn, or rotate the device.

7. Confirm the positioning of the device relative to the clot. Unsheathe the baskets and continue to retract the microcatheter to ensure that the baskets remain distal to the microcatheter tip while retracting the device and microcatheter.

Clasping the Thrombus

1. Press the thumb grip to unlock the Handle and slowly slide the thumb grip proximally to pull the distal basket toward the proximal basket.
2. Observe the proximal and distal basket gap shortening.
3. Release the thumb grip to lock and maintain this position on the Handle and maintain the clasp on the thrombus.

Note: If significant movement between the proximal and distal baskets was not visualized, check the following:

- Under fluoroscopy look for any proximal kinking in the guide catheter, intermediate catheter, or microcatheter.
- Gently apply forward tension to the hypotube or backward tension on the microcatheter, or both, while observing on fluoroscopy for any delayed proximal basket movement.

Thrombus Retrieval

1. Lock the microcatheter RHV onto the device.
2. With the Handle locked, while holding the guide catheter stationary with one hand, slowly and gently initiate movement of the device and microcatheter by retracting them. Continue pulling the device and microcatheter back steadily to withdraw the device and microcatheter together. Observe the thrombus retrieval on fluoroscopy.

Note: If there is excessive resistance while withdrawing the ThrombX Retriever into the tip of the guide catheter, lengthen the distance between the baskets by shortening the handle gap. If excessive resistance persists while withdrawing the ThrombX Retriever into the tip of the guide catheter, remove the guide catheter and ThrombX Retriever together as a unit into the introducer sheath.

3. Open the guide catheter RHV to allow the device to be withdrawn without resistance, using care to avoid introduction of air.
4. Aspirate the guide catheter when the device is pulled into and through the guide catheter.
5. Loosen the RHV to allow the microcatheter and the ThrombX Retriever to exit without resistance.
6. After removal of the ThrombX Retriever, aspirate the guide catheter adequately to ensure it is free of thrombus.
7. Perform a contrast injection to assess flow.

Preparation for Redeployment

1. If the device is to be redeployed between thrombectomy attempts, separate the baskets and gently remove any clot. Rinse the baskets as necessary and verify that there is no damage to the baskets (replace device if there is damage). Remove the Handle from the device by unscrewing both caps.
2. Obtain the Adapter (remove from the packaging hoop) and Introducer from the sterile field.
3. Insert the Introducer into the Luer end of Adapter. Backload the proximal wire of the device into the Adapter. Feed the device through the Adapter and Introducer. Pull the baskets into the Adapter first, followed by the Introducer, keeping the Adapter on the Introducer.
4. Confirm that the baskets are not overlapped.
5. Replace the handle by inserting it onto the proximal end of the device until the inner pusher wire abuts the cap, as seen in the window. Ensure the handle remains in the locked position during handle reattachment. A physical "stop" will be felt when the pusher wire abuts the end of the proximal cap.
6. Tighten the proximal cap (hold the slider with one hand while tightening the knob with the other hand).
7. Repeat the steps from the beginning of the **Procedure** section.

Caution: The ThrombX Retriever should not be deployed more than three times during the procedure.

Warranty

ThrombX Medical, Inc. warrants that this medical device is free from defects in both materials and workmanship. Any other express or implied warranties, including warranties of merchantability or fitness, are hereby disclaimed. Suitability for use of this medical device for any particular surgical procedure should be determined by the user in conformance with the manufacturer's instructions for use. ThrombX will not be responsible for any product that is re-sterilized, nor accept for credit or exchange any product that has been opened but not used. If the inner unit is not opened or damaged, the product is sterile and pyrogen free. **There are no warranties that extend beyond the description on the face hereof.**
